# LDLR H3K27ac in PBMCs: An Early Warning Biomarker for Hypercholesterolemia Susceptibility in Male Newborns Treated with Prenatal Dexamethasone

**DOI:** 10.3390/toxics13080651

**Published:** 2025-07-31

**Authors:** Kexin Liu, Can Ai, Dan Xu, Wen Hu, Guanghui Chen, Jinzhi Zhang, Ning Zhang, Dongfang Wu, Hui Wang

**Affiliations:** 1 Department of Pharmacology, School of Basic Medical Science, Wuhan University, Wuhan 430071, China; kathiehubu@163.com (K.L.); candyaican@foxmail.com (C.A.); xuyidan70188@163.com (D.X.); chen_guanghui@whu.edu.cn (G.C.); 2 Department of Pharmacy, Zhongnan Hospital of Wuhan University, Wuhan 430072, China; huwen0113@163.com; 3 Hubei Provincial Key Laboratory of Developmentally Originated Disorder, Wuhan 430071, China

**Keywords:** dexamethasone, epigenetic inheritance, hypercholesterolemia, peripheral blood mononuclear cells, warning biomarker

## Abstract

Dexamethasone, widely used as an exogenous glucocorticoid in clinical and animal practice, has recently been recognized as an environmental contaminant of concern. Existing evidence documents its ability to induce persistent dyslipidemia in adult offspring. In this study, plasma cholesterol levels in male rats exposed to dexamethasone prenatally (PDE) were increased. Meanwhile, developmental tracking revealed a reduction in hepatic low-density lipoprotein receptor (LDLR) promoter H3K27 acetylation (H3K27ac) and corresponding transcriptional activity across gestational-to-postnatal stages. Mechanistic investigations established glucocorticoid receptor/histone deacetylase2 (GR/HDAC2) axis-mediated epigenetic programming of LDLR through H3K27ac modulation in PDE offspring, potentiating susceptibility to hypercholesterolemia. Additionally, in peripheral blood mononuclear cells (PBMC) of PDE male adult offspring, LDLR H3K27ac level and expression were also decreased and positively correlated with those in the liver. Clinical studies further substantiated that male newborns prenatally treated with dexamethasone exhibited increased serum cholesterol levels and consistent reductions in LDLR H3K27ac levels and corresponding transcriptional activity in PBMC. This study establishes a complete evidence chain linking PDE with epigenetic programming and cholesterol metabolic dysfunction, proposing PBMC epigenetic biomarkers as a novel non-invasive monitoring tool for assessing the developmental toxicity of chemical exposures during pregnancy. This has significant implications for improving environmental health risk assessment systems.

## 1. Introduction

Dexamethasone, the most widely used synthetic glucocorticoid, is extensively used in clinical settings owing to its demonstrated efficacy in reducing respiratory distress syndrome morbidity and mortality rates. Current clinical data indicate its utilization in over 60% of neonatal intensive care scenarios, reflecting the established therapeutic protocols [1]. Furthermore, dexamethasone is widely misused in the livestock industry due to its growth-promoting effects. Surveys indicate that in numerous farms in Sicily, the concentration of dexamethasone in bovine liver samples often exceeds the maximum residue limit, reaching up to 1.8 μg/kg [2]. The multimodal environmental dissemination of dexamethasone has resulted in its progressive environmental ubiquity, with analytical quantification verifying its occurrence across aquatic matrices (surface/river waters, wastewater), sedimentary deposits, terrestrial soils, and potable water supplies. The measured concentrations span nearly five orders of magnitude (0.02–3770 ng/L), demonstrating extensive contamination gradients [3,4]. This indicates that dexamethasone is an emerging environmental pollutant.

Emerging evidence from developmental toxicology research has established that prenatal dexamethasone exposure (PDE) may lead to metabolic disorders in offspring, particularly lipid metabolism abnormalities. Specifically, PDE induces persistent elevations in circulatory total cholesterol (TCH) and low-density lipoprotein cholesterol (LDL-C) in offspring, substantiating its role in dyslipidemia programming [5,6,7]. Our team developed a validated PDE animal model demonstrating dexamethasone-induced sustained hypercholesterolemia in adult male progeny, mechanistically linked to in utero hepatic cholesterol metabolic reprogramming [8], indicating its origin in intrauterine life. Emerging evidence indicates that gestational insults program persistent hypercholesterolemia through epigenetic mediators [9]. Mechanistic studies have demonstrated that glucocorticoid exposure alters the histone modification landscapes of metabolic genes across biological systems [10]. Therefore, we hypothesized that PDE-induced hypercholesterolemia exerts cross-organ effects through epigenetic programming. This suggests that the epigenetic features of these diseases hold great potential for biomarker development. Although studies have explored how various environmental factors can trigger hypercholesterolemia by altering fetal cholesterol metabolism [9], there is still a lack of effective biomarkers and risk assessment systems, leaving a gap in early clinical warning technologies. Consequently, the development of early warning technologies against environmental pollution is crucial for effectively mitigating metabolic disorders in susceptible populations.

Peripheral blood mononuclear cells (PBMCs) are cells in the peripheral blood that have a single nucleus, primarily composed of lymphocytes and a small number of monocytes, making them an easily accessible biological sample in clinical settings. Emerging evidence reveals that dysregulated epigenetic programming in PBMCs during early life predicts adult-onset disease susceptibility [11,12]. This evidence drives international efforts to identify circulating warning biomarkers for fetal-origin diseases. Experimental models have demonstrated that PDE-induced cyclin-dependent kinase 5 (CDK5) expression patterns in PBMCs correlate with hippocampal CDK5 activity, providing quantifiable indicators of offspring neurocognitive dysfunction [10]. These discoveries establish a framework for early toxicity alert systems utilizing developmental epigenomic signatures. However, a critical, unexplored question remains: Does PDE-induced disruption of hepatic cholesterol homeostasis in offspring involve dysregulated histone acetylation at sterol-regulatory genes, potentially generating translational epigenetic biomarkers in PBMCs for predicting developmental-originated dyslipidemia susceptibility? Currently, there is a paucity of relevant research in this field.

This study aims to explore the mechanism of susceptibility to hypercholesterolemia in male offspring caused by dexamethasone exposure during pregnancy and to investigate its novel early warning biomarker. This study confirmed the onset of PDE-induced hypercholesterolemia in adult male offspring rats. Subsequently, integrating findings from animal experiments and human fetal liver cell line studies, we investigated the intrauterine epigenetic programming mechanisms and biomarkers related to hepatic cholesterol metabolism. Lastly, by employing multi-indicator testing and correlation analysis of liver and PBMCs from male offspring rats, as well as PBMCs from clinical male neonates, we sought to identify early warning biomarkers for fetal-originated hypercholesterolemia susceptibility. This research not only deepens the understanding of dexamethasone-induced hepatic developmental toxicity but also presents the potential for establishing early warning technologies for fetal hypercholesterolemia. It aims to facilitate the translation from basic research to clinical application and provide scientific evidence for environmental health risk assessment and management.

## 2. Materials and Methods

### 2.1. Chemicals and Reagents

The sources of the chemicals and reagents used are listed in Table S1.

### 2.2. Animals and Treatment

Specific pathogen-free (SPF) Wistar rats (200–240 g for females and 260–300 g for males) were obtained from the Experimental Center of Hubei Medical Scientific Academy (No. 2017-0018, Wuhan, China). Animal experiments were performed at the Center for Animal Experiments of Wuhan University (Wuhan, China), which is accredited by the Association for Assessment and Accreditation of Laboratory Animal Care International (AAALAC International). All experimental animal procedures were approved and performed in accordance with the Guidelines for the Care and Use of Laboratory Animals of the Chinese Animal Welfare Committee. The Committee on the Ethics Committee of the Animal Experiment Center of Wuhan University approved the protocol (Permit No. 201719).

Animals were housed under standard conditions (room temperature: 18–22 °C; humidity: 40–60%) and were allowed free access to rat chow and tap water. After one week of acclimation, the animals were bred and mated as previously described [8]. The day of vaginal smear confirmation of mating was designated GD0. Pregnant rats were transferred to individual cages and then randomly divided into control and PDE groups. Every morning from GD9 to GD20, the rats in the PDE group were injected subcutaneously with dexamethasone (0.2 mg/kg·d), and the control group received the same volume of saline solution. On GD20, a subset of pregnant rats was anesthetized under 3% isoflurane and then sacrificed in a room separate from the other animals. Pregnant rats with litter sizes between 8 and 14 pups were considered qualified for further experiments. The fetal livers were collected, immediately frozen in liquid nitrogen, and stored at −80 °C for subsequent analyses.

Another set of pregnant rats in the control and PDE groups (*n* = 12 per group) was maintained until normal delivery. On postnatal day (PD) 1, the number of 8 pups per litter was normalized to eight to ensure a balanced diet. After weaning (PW4), the pups were fed lab chow. At PW8 and PW28, the male rats were anesthetized under 3% isoflurane to collect blood and liver samples, which were then frozen in liquid nitrogen and stored at −80 °C for subsequent analyses (Figure S1).

### 2.3. L02 Cell Culture and Treatment

The human L02 cell line was purchased from the Wuhan University Deposit Center (Wuhan, China). The cells were cultured in DMEM medium (Gibco, Grand Island, CA, USA) supplemented with 10% fetal bovine serum and antibiotics (100 unit/mL penicillin and 100 μg/mL streptomycin) in a 5% CO_2_ humidified incubator (Thermo, Waltham, MA, USA) at 37 °C. The cells were treated with various concentrations of dexamethasone (0, 100, 500, and 2500 nM) for 24 h and then harvested for subsequent analyses. The cytotoxicity of dexamethasone was determined using MTS. Water-soluble cholesterol (25 μg/mL in methyl-beta cyclodextrin) was administered, and LDLR was overexpressed to confirm that reduced LDLR expression mediated dexamethasone-induced extracellular cholesterol increase. To confirm the role of GR and HDAC2 in the gene regulation of dexamethasone, the cells were incubated with dexamethasone (500 nM) and/or glucocorticoid receptor (GR) antagonist RU486 (10 nM) or the HDAC2 inhibitor Santacruzamate A (120 nM) for further analyses.

### 2.4. Co-Immunoprecipitation

Co-immunoprecipitation (Co-IP) of proteins was performed using Protein G Magnetic Beads (Millipore, Bedford, MA, USA) following the manufacturer’s instructions. After several washes with ice-cold PBS, 1 mL of pre-cooled lysis buffer was added to the cell culture flask for 30 min. The suspension was then centrifuged at 2000× *g*, 4 °C for 15 min. After that, 50 μL of the supernatant was used as input protein, and the other was divided into 400 μL per Eppendorf tube and added with 1 μg GR or HDAC2 antibody or a normal IgG of the same species as the negative control and incubated overnight at 4 °C. After that, 40 μL of Protein G Magnetic Beads was added to the above mixture and incubated for 1 h at 4 °C. The bead–antibody–antigen complexes were collected by centrifugation and washed three times with lysis buffer. Then, 30 μL loading buffer was added to each sample, followed by a water bath at 100 °C. Western blotting was used to detect protein levels.

### 2.5. Chromatin Immunoprecipitation (ChIP) and Re-ChIP Assay

ChIP assay was performed on liver tissues and L02 cells to evaluate the levels of GR-HDAC2 binding and acetylation of histone 3 lysine 9, 14, and 27 (H3K9ac, H3K14ac, and H3K27ac) at the promoter region of LDLR (rat and human) gene according to a modified version of the manufacturer’s protocol. The samples were cross-linked with 1% formaldehyde on a rocker for 10 min, and 125 mM glycine was added to stop the reaction. The samples were then centrifuged and resuspended in 0.5 mL of lysis buffer containing protease inhibitors. The cell lysates were sonicated to shear DNA to approximately 200 bp and transferred to a new tube with ChIP dilution buffer. Chromatin was incubated overnight at 4 °C on a nutator/rocker with a specific antibody (diluted 1:50) for GR, HDAC2, H3K9ac, H3K14ac, H3K27ac, IgG, and BSA-treated Protein G beads to reduce nonspecific background binding. The immunoprecipitated DNA-protein complex with beads was collected by centrifugation and washed sequentially with low-salt, high-salt, LiCl immune complex, and Tris-EDTA washing buffer solutions. A freshly prepared elution buffer containing 1% SDS and 0.1 M NaHCO3 was used to elute the DNA-protein complexes. The samples were then placed in 65 °C water baths overnight to reverse formaldehyde cross-linking and subsequently purified using a DNA purification kit. For re-ChIP, the beads were eluted with 20 mM dithiothreitol at 37 °C for 30 min, and the eluates were diluted 30-fold for further incubation with the appropriate secondary antibody (HDAC2 or GR) and the beads. Gene enrichment was quantified relative to the input controls by qPCR using primers specific to the promoter regions. The primers used are listed in Table S2.

### 2.6. Luciferase Activity Assay

The luciferase reporter plasmids carrying the wildtype or mutated (MUT) HDAC2 or LDLR promoter (PGL3-HDAC2, PGL3-HDAC2mut, PGL3-LDLR, and PGL3-LDLRmut) were transfected into L02 cells along with GR-overexpressed plasmids (0, 50, 100, and 200 ng) using Lipofectamine 3000. After transfection (24 h), the cells were lysed, and luciferase activity was measured using the Dual-Luciferase Reporter Assay System.

### 2.7. Human Subjects, Blood Collection, Plasma, and PBMC Isolation

The human study was approved by the Ethics Committee of the Medical College of Wuhan University, China (Permit No. 201819). A cross-sectional study was conducted using the medical records of pregnant women and their neonates from 1 October 2018 to 30 January 2019. The following criteria were used for inclusion: ① Neonates who need admission to the neonatal intensive care unit; ② Pregnancies who have received treatment of dexamethasone between 34 to 42 weeks of gestation; or ③ Received treatment of dexamethasone at 23 to 33 6/7 weeks but delivered beyond 24 h to 7 days; or ④ Those who delivered between 23 to 42 weeks without administration of dexamethasone. The 60 male neonates recruited for this study were categorized into two groups: the control group with no dexamethasone administration and the dexamethasone group.

Demographic and pregnancy characteristics of the recruited subjects were collected. Detailed information is provided in Table S3. We compared maternal age, gestational age, birth weight of neonates, and Apgar scores at 1 min and 5 min between the two groups. The Apgar score can describe the condition of a newborn and, when properly applied, can be used as a tool to predict infant outcomes. To avoid confusion regarding the diseases and multiple pregnancies in the results, pregnant women with abnormal liver function and hereditary metabolic diseases were excluded. To avoid artifacts from the effects of labor, only blood samples obtained from neonates who underwent cesarean sections were selected. All donors or their guardians provided written consent, and ethical permission was obtained to use all samples. Blood samples (2–3 mL) were obtained using ethylenediaminetetraacetic acid (EDTA) vacutainers within 72 h after birth. After routine blood tests, PBMCs were immediately isolated from the remaining blood using Ficoll-Paque density gradient medium. All analyses described below were performed on individual samples.

The methods used for TCH, HDL-C, and LDL-C concentration detection, total RNA extraction, reverse transcription, RT-qPCR, western blotting, and immunofluorescence analysis are shown in the Supplementary Materials.

### 2.8. Statistical Analysis

SPSS 19 (SPSS Science Inc., Chicago, IL, USA) and Prism 8.0 (GraphPad Software, La Jolla, CA, USA) were used for data analysis. Quantitative data were expressed as scatter plots with bars (histogram plus scatter with SD). All samples were biological replicates. The Shapiro−Wilk test was employed to examine the normality of the data. Homogeneity of variances was evaluated using Levene’s test. After testing for normality and homogeneity of variance, the data of experiments with multiple groups were analyzed using one-way analysis of variance (ANOVA) with Tukey’s post hoc test for multiple comparisons. Clinical data were analyzed using parameter estimates with 95% confidence intervals (CI), and Cohen’s d was used to quantify effect sizes. Statistical significance was defined as a two-sided alpha level of 0.05. Data from experiments between the two groups were compared using Student’s *t*-test. Statistical significance was set at *p* < 0.05.

## 3. Results

### 3.1. Intrauterine Origin and Toxic Target of Hypercholesterolemia in Male PDE Adult Offspring Rats

#### 3.1.1. Lowered Cholesterol Levels During Intrauterine Period and Postnatal Hypercholesterolemia in Male PDE Offspring Rats

**Figure 1:**
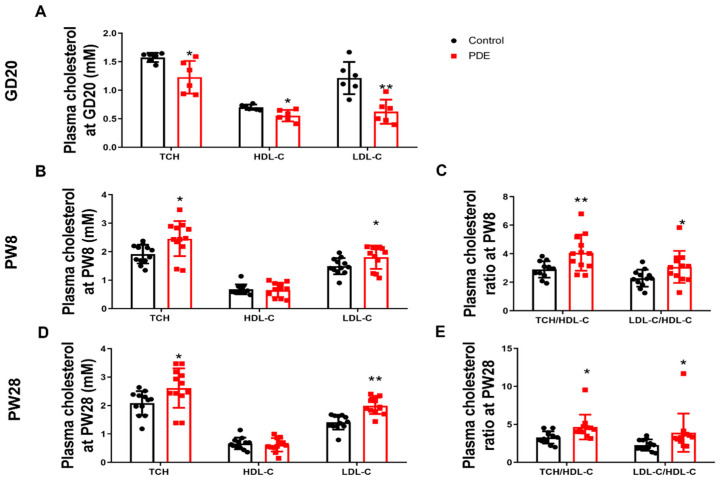
Effects of prenatal dexamethasone exposure (PDE) on plasma cholesterol phenotypes in rat offspring. (**A**,**B**,**D**) Plasma levels of total cholesterol (TCH), high-density lipoprotein cholesterol (HDL-C), and low-density lipoprotein cholesterol (LDL-C) on gestational day (GD) 20, postnatal day (PW) 8, and PW28. (**C**,**E**) Plasma levels of the TCH/HDL-C and LDL-C/HDL-C ratios at PW8 and PW28. Mean ± SD. *n* = 6 for the intrauterine period and 12 for after birth. Student’s *t*-tests were used to compare the control and PDE group differences. * *p* < 0.05, ** *p* < 0.01 vs. control group.

We first analyzed the impact of PDE (0.2 mg/kg·d subcutaneously administered from GD9-20) on the cholesterol profiles of male offspring. At GD20, the PDE group demonstrated significantly lower TCH, HDL-C, and LDL-C levels compared to controls (*p* < 0.05, *p* < 0.01, Figure 1A). Postnatal assessments revealed elevated TCH and LDL-C concentrations at PW8 (*p* < 0.05) and PW28 (*p* < 0.05, *p* < 0.01), while HDL-C levels showed no significant changes (Figure 1B,D). Cardiovascular and cerebrovascular risks were evaluated using atherogenic indices (TCH/HDL-C and LDL-C/HDL-C ratios) [13,14]. The PDE group exhibited higher ratios than the controls at both PW8 and PW28 (*p* < 0.05, *p* < 0.01, Figure 1C,E). These results indicate that PDE induces intrauterine cholesterol suppression, followed by postnatal hypercholesterolemia, potentially elevating the risk of cardiovascular disease.

#### 3.1.2. H3K27ac-LDLR Epigenetic and Transcriptional Regulation in PDE Male Offspring Rats

**Figure 2:**
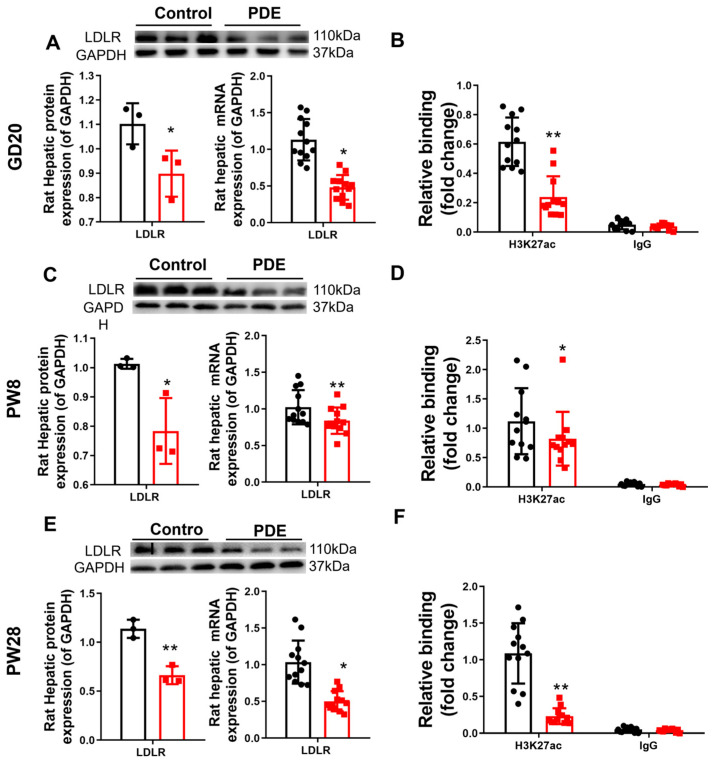
Effects of prenatal dexamethasone exposure (PDE) on hepatic low-density lipoprotein receptor (LDLR)and histone 3 lysine 27 acetylation (H3K27ac) levels in fetal and adult offspring rats. (**A**,**C**,**E**) mRNA and protein expression of hepatic LDLR at gestational day (GD) 20, postnatal day (PW) 8, and PW28. (**B**,**D**,**F**) Levels of H3K27ac at the promoter region of hepatic LDLR at GD20, PW8, and PW28. *n* = 12 for mRNA expression and chromatin immunoprecipitation (ChIP)-PCR, *n* = 3 for protein expression. Mean ± SD. Student’s *t*-tests were used to compare the differences between the control and PDE groups. * *p* < 0.05, ** *p* < 0.01 vs. control group.

To elucidate the intrauterine origin mechanism causing increased plasma cholesterol phenotypes by PDE, we examined alterations in the expression of the hepatic cholesterol biosynthesis-related transcription factor SREBP2, synthase HMGCR, output-related carrier protein ApoB, and reverse transport-related receptors (SR-B1 and LDLR) across various developmental stages. Our data revealed sustained downregulation of LDLR in male PDE offspring, with both mRNA and protein expression showing significant reductions at GD20, PW8, and PW28 compared to controls (*p* < 0.01, *p* < 0.05, Figure 2A,C,E). The expression levels of SREBP2, HMGCR, ApoB, and SR-B1 are shown in Figure S2. At GD20, decreased expression of SREBP2, HMGCR, ApoB, and SR-B1 was observed at the transcriptional and translational levels. The expressions of PW8, SREBP2, and ApoB were upregulated while HMGCR and SR-B1 remained unaffected. At PW28, HMGCR and SR-B1 expression declined significantly, in contrast to the stable SREBP2/ApoB levels. Histone modification analysis identified specific H3K27ac depletion (rather than H3K9ac and H3K14ac, as shown in Figure S3) at the LDLR promoter in the PDE groups. This epigenetic alteration persisted both prenatally and postnatally (*p* < 0.05, *p* < 0.01, Figure 2B,D,F). This finding confirms persistent LDLR suppression through H3K27ac reduction in male PDE offspring across developmental stages.

### 3.2. Developmental Epigenetic Programming of Hypercholesterolemia in Male PDE Offspring Rats

#### 3.2.1. Hepatic GR/HDAC2 Signaling and LDLR Transcriptional Patterns in Male PDE Offspring Rats

**Figure 3:**
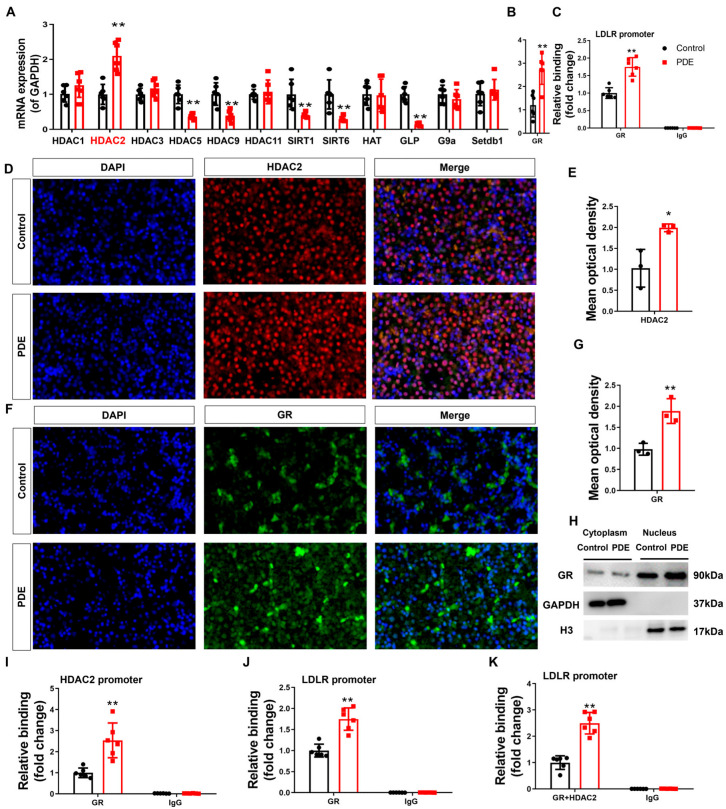
Glucocorticoid receptor-histone deacetylase 2 (GR-HDAC2) participates in the inhibition of hepatic low-density lipoprotein receptor (LDLR) expression caused by prenatal dexamethasone exposure (PDE). (**A**,**B**) mRNA expression of hepatic histone modification enzymes and GR. (**C**) Binding of hepatic HDAC2 to the LDLR promoter through chromatin immunoprecipitation (ChIP)-PCR. (**D**–**G**) Protein expression of hepatic HDAC2 and GR was measured by immunofluorescence. (**H**) Protein levels of hepatic GR in the cytoplasm and nucleus, as determined by Western blotting. (**I**,**J**) Binding of hepatic GR at HDAC2 and LDLR promoters as determined by ChIP-PCR. (**K**) Binding of hepatic GR and HDAC2 complex at LDLR promoter through re-ChIP-PCR. *n* = 12 for mRNA expression and ChIP-PCR, *n* = 3 for protein expression. Mean ± SD. Student’s *t*-tests were used to compare the control and PDE group differences. * *p* < 0.05, ** *p* < 0.01 vs. control group.

To understand the intrauterine origin basis of PDE-d-induced H3K27ac depletion at the LDLR promoter in male offspring rats, we first identified potential epigenetic enzymes involved in regulating fetal hepatic epigenetic modifications. Our data demonstrated significantly elevated HDAC2 mRNA and protein levels in the fetal livers of male PDE offspring compared to controls (*p* < 0.01, *p* < 0.05, Figure 3A,D,E). Enhanced HDAC2 binding to the LDLR promoter was confirmed by ChIP assays (*p* < 0.01, Figure 3C), indicating PDE-induced HDAC2 upregulation and promoter interactions. GR analysis in the fetal liver revealed increased mRNA expression (*p* < 0.01, Figure 3B), protein expression, nuclear translocation (*p* < 0.01, Figure 3F–H), and strengthened binding to both HDAC2 and the LDLR promoter (*p* < 0.01, Figure 3I,J) in the PDE groups. Re-ChIP validated intensified co-binding of GR/HDAC2 at the LDLR promoter (*p* < 0.01, Figure 3K), demonstrating PDE’s ability to amplify GR-HDAC2 collaboration at cholesterol regulatory loci.

#### 3.2.2. GR Activation Drives Dexamethasone-Induced LDLR Suppression and Extracellular Cholesterol Accumulation via Dual Mechanisms

**Figure 4:**
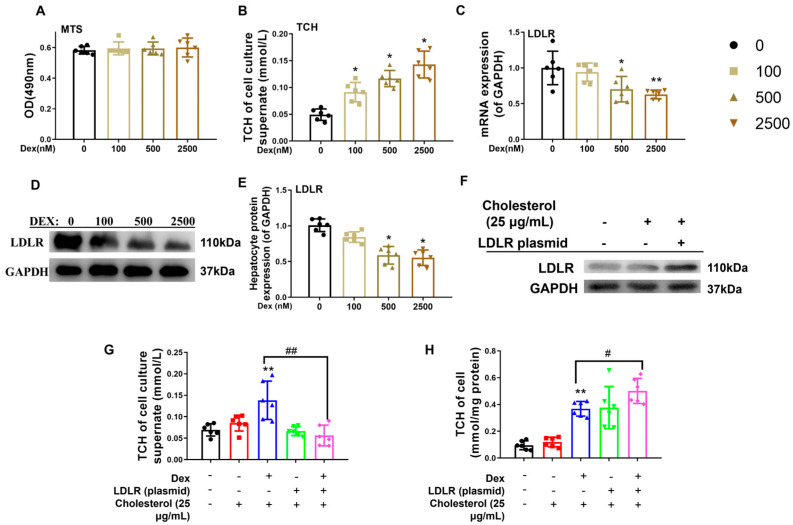
Low-density lipoprotein receptor (LDLR) mediated the changes in cholesterol levels in L02 cells induced by dexamethasone. (**A**) Optical density (OD) value by the 3-(4,5-dimethylthiazol-2-yl)-5-(3-carboxymethoxyphenyl)-2-(4-sulfophenyl)-2H-tetrazolium, inner salt (MTS) analysis. (**B**) Total cholesterol (TCH) levels in cell culture supernatants treated with different doses of dexamethasone for 24 h. (**C**) mRNA expression of cholesterol metabolism genes, such as sterol regulatory element binding protein 2 (SREBP2), HMG CoA reductase (HMGCR), apolipoprotein B (ApoB), scavenger receptor B1 (SR-B1), and LDLR, treated with different doses of dexamethasone for 24 h. (**D**,**E**) Protein expression of cholesterol metabolism genes, such as HMGCR, SREBP2, ApoB, SR-B1, and LDLR after treatment with different doses of dexamethasone for 24 h. (**F**) Protein expression of LDLR treated with LDLR plasmid or 25 μg/mL of cholesterol. (**G**) TCH levels in culture supernatant treated with 25 μg/mL cholesterol, 500 nM dexamethasone, and/or LDLR plasmid for 24 h. (**H**) TCH levels in L02 cells treated with 25 μg/mL cholesterol, 500 nM dexamethasone, and/or LDLR plasmid for 24 h. *n* = 6. Mean ± SD.One-way ANOVA with post-hoc tests was used to analyze multiple group comparisons. * *p* < 0.05, ** *p* < 0.01 vs. control groups; ^#^ *p* < 0.05, ^##^ *p* < 0.01 vs. corresponding interference group.

To assess dexamethasone-mediated modulation of hepatic cholesterol homeostasis, mature L02 hepatocytes (a well-established fetal pathophysiological model) were exposed to gradient doses of dexamethasone. Experimental data demonstrated that 24 h exposure to 100–2500 nM dexamethasone maintained normal cell viability (Figure 4A), dose-dependently elevated extracellular cholesterol concentrations (*p* < 0.05, Figure 4B), and suppressed LDLR expression at both the transcriptional and translational levels (*p* < 0.05, *p* < 0.01, Figure 4C–E). To establish causality between LDLR downregulation and dexamethasone-induced cholesterol dysregulation, rescue experiments were conducted using a 25 μg/mL water-soluble cholesterol complex combined with 500 nM dexamethasone and/or an LDLR overexpression vector [8]. Notably, LDLR reconstitution effectively counteracted dexamethasone-mediated elevation of extracellular cholesterol (*p* < 0.01, Figure 4F,G) while restoring intracellular cholesterol homeostasis (*p* < 0.05, *p* < 0.01, Figure 4H), confirming that dexamethasone-induced LDLR suppression disrupts cholesterol transport mechanisms, culminating in elevated extracellular cholesterol accumulation.

**Figure 5:**
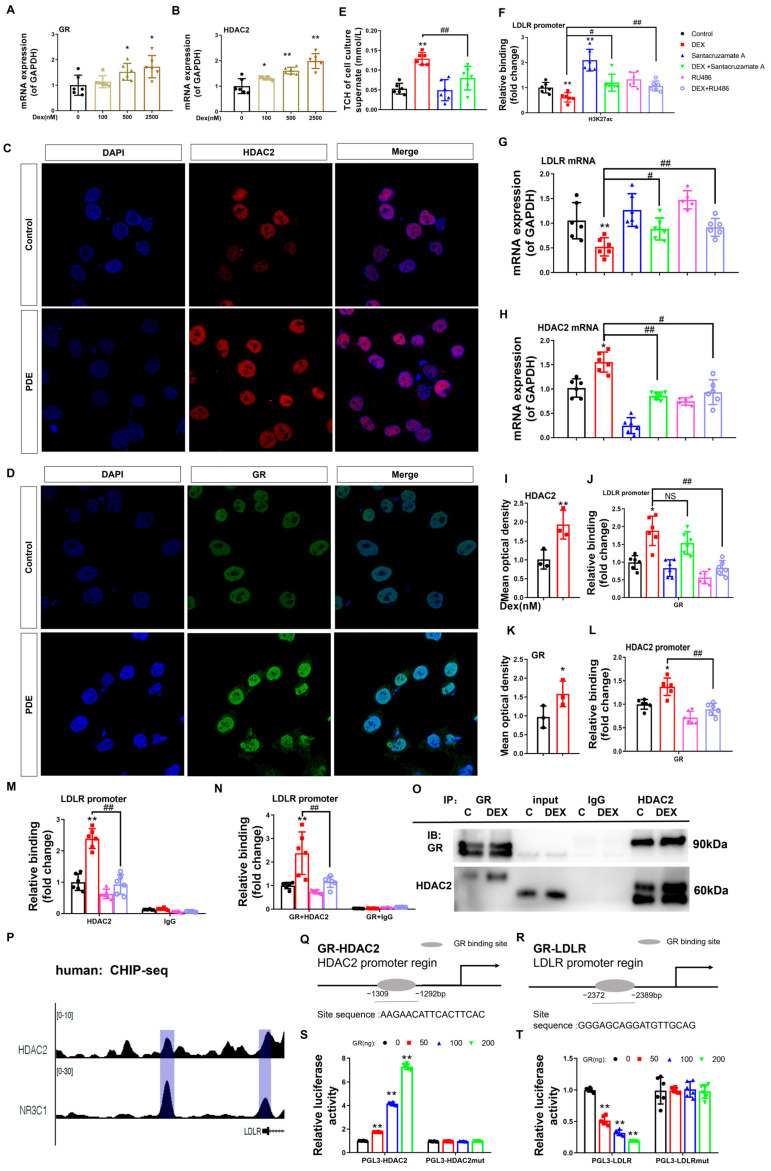
Glucocorticoid receptor-histone deacetylase 2 (GR-HDAC2) signaling mediates the dexamethasone-induced reduction in low-density lipoprotein receptor (LDLR) histone 3 lysine 27 acetylation (H3K27ac) and mRNA expression levels in L02 cells. (**A**,**B**) mRNA expression of GR and HDAC2. (**C**,**D**,**I**,**K**) Protein expression of HDAC2 and GR was determined by immunofluorescence. (**E**) H3K27ac levels at the LDLR promoter. (**F**) mRNA expression of LDLR. (**G**) Total cholesterol (TCH) levels in the cell culture supernatant. (**H**) GR binding to the promoter of LDLR. (**J**) mRNA expression of HDAC2. (**L**) GR binding to the promoter of HDAC2. (**M**) HDAC2 binding to the promoter of LDLR. (**N**) GR and HDAC2 binding to the promoter of LDLR as determined by re-ChIP. (**O**) GR and HDAC2 binding by Co-IP analysis. (**P**) Analysis of GR (NR3C1) and HDAC2 at the LDLR promoter by ChIP-seq. (**Q**) Glucocorticoid-responsive element (GRE) in the human HDAC2 promoter region. (**R**) GRE in the human LDLR promoter region. (**S**) Luciferase activity of HDAC2 when transfected with HDAC2-GRE and different concentrations of GR plasmids. (**T**) Luciferase activity of LDLR when transfected with LDLR-negative glucocorticoid responsive element (nGRE) and different concentrations of GR plasmid. *n* = 6. Mean ± SD. One-way ANOVA with post-hoc tests was used to analyze multiple group comparisons. * *p* < 0.05, ** *p* < 0.01 vs. control groups; ^#^ *p* < 0.05, ^##^ *p* < 0.01 vs. corresponding interference group.

To verify the epigenetic basis of dexamethasone-mediated LDLR suppression in fetal hepatocytes, pharmacological interventions were implemented using Santacruzamate A (an HDAC2-selective inhibitor) and RU486 (a GR antagonist). Quantitative analysis revealed a dose-responsive elevation of GR and HDAC2 mRNA levels in L02 hepatocytes treated with dexamethasone compared to untreated controls (*p* < 0.05, *p* < 0.01, Figure 5A,B). Nuclear protein quantification confirmed concurrent increases in both GR and HDAC2 (*p* < 0.05, *p* < 0.01, Figure 5C,D,I,K). Dexamethasone exposure reduced H3K27ac histone modification at the LDLR promoter, correlating with suppressed LDLR expression and elevated extracellular cholesterol levels (*p* < 0.05, Figure 5E–G). Co-treatment with Santacruzamate A effectively counteracted dexamethasone-induced reductions in promoter H3K27ac, restored LDLR expression, and normalized extracellular cholesterol compared to the dexamethasone-only group (*p* < 0.05, Figure 5E–G). This pharmacological evidence establishes HDAC2-dependent H3K27 deacetylation as the principal epigenetic mechanism by which dexamethasone disrupts cholesterol transport via LDLR suppression.

Furthermore, ChIP quantification revealed dexamethasone-potentiated GR-DNA interactions at the HDAC2 and LDLR transcriptional start sites (*p* < 0.05, *p* < 0.01, Figure 5H,L). Simultaneously, dexamethasone administration enhanced HDAC2 chromatin recruitment to LDLR regulatory elements (*p* < 0.05, *p* < 0.01, Figure 5M), demonstrating tripartite chromatin interactions in the regulation of cholesterol homeostasis. Co-IP assays confirmed dexamethasone-enhanced GR-HDAC2 protein interaction (Figure 5O). RU486 treatment reversed these effects and normalized HDAC2 mRNA levels (*p* < 0.05, *p* < 0.01, Figure 5E,F,H,J,L,M). Re-ChIP analysis demonstrated GR-driven HDAC2 recruitment to the LDLR promoter (*p* < 0.01, Figure 5N), while RU486 treatment reversed this effect (*p* < 0.01, Figure 5N). ENCODE database mining of GR (NR3C1) and HDAC2 ChIP-seq datasets (Figure 5P) mapped GR occupancy peaks at human HDAC2 (−1309 to −1292 bp) and LDLR (−2372 to −2389 bp) promoter loci (Figure 5Q,R). Functional validation in L02 hepatocytes was performed using dual transfection with GR plasmid gradients and HDAC2/LDLR promoter-driven luciferase reporters (wild-type vs. mutated variants). We found that the fluorescence activity of wild-type PGL3-HDAC2 (or PGL3-LDLR) increased (or decreased) in a concentration-dependent manner with GR transfection (*p* < 0.01, Figure 5S,T), while the fluorescence activity of the mutated PGL3-HDAC2mut (or PGL3-LDLRmut) did not change. This indicates that GR can directly bind to the HDAC2 and LDLR promoter regions, reducing (or increasing) the transcriptional activity of the HDAC2 (or LDLR) promoter.

In conclusion, dexamethasone stimulated GR activation and translocation to the nucleus in hepatocytes. This process has two main effects. First, GR directly binds to the negative glucocorticoid response element (nGRE) within the LDLR promoter region to downregulate its transcriptional activity. Second, GR interacts with the glucocorticoid response element (GRE) of the HDAC2 promoter to enhance HDAC2 expression, while simultaneously recruiting HDAC2 protein to cooperatively target the nGRE site on the LDLR promoter. This synergistic interaction amplifies the suppression of LDLR expression through the HDAC2-mediated reduction of H3K27ac levels in the LDLR promoter region.

### 3.3. Animal and Human Studies Identified Peripheral Biomarkers Predicting Male Offspring Hypercholesterolemia Risk

#### 3.3.1. PBMC-Liver Epigenetic Exhibited Conserved Correlation in Male Offspring

**Figure 6:**
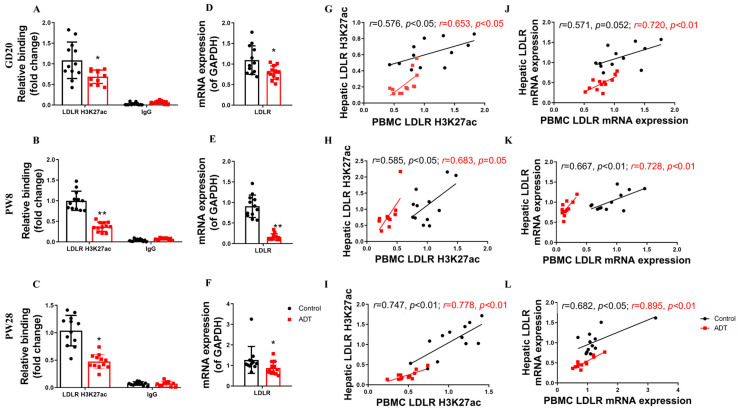
The low-density lipoprotein receptor (LDLR) histone 3 lysine 27 acetylation (H3K27ac) and mRNA expression levels in peripheral blood mononuclear cells (PBMC) and their correlation with hepatic LDLR H3K27ac and mRNA expression levels in male offspring rats by prenatal dexamethasone exposure (PDE). (**A**–**C**) H3K27ac levels in the LDLR promoter in PBMC on gestational day (GD) 20, postnatal day (PW) 8, and PW28. (**D**–**F**) LDLR mRNA expression in PBMC at GD20, PW8, and PW28. (**G**–**I**) Correlation of H3K27ac levels in the LDLR promoter between PBMC and the liver at GD20, PW8, and PW28. (**J**–**L**) Correlation of LDLR mRNA expression between PBMC and liver at GD20, PW8, and PW28. *n* = 12. Mean ± SD. Student’s *t*-tests were used to compare the control and PDE group differences. * *p* < 0.05, ** *p* < 0.01 vs. control group.

To investigate the translational potential of circulating biomarkers for hypercholesterolemia predisposition in PDE offspring, we conducted a comparative analysis of LDLR promoter H3K27ac epigenetic signatures in PBMC and hepatic LDLR transcriptional activity across antenatal and postnatal developmental stages in male offspring. The experimental results demonstrated significant epigenetic and physiological alterations in the PDE group. Compared with the controls, male PDE offspring showed marked reductions in PBMC LDLR promoter H3K27ac levels and corresponding gene expression at GD20, PW8, and PW28 (*p* < 0.05, *p* < 0.01, Figure 6A–F). These findings confirm consistent PBMC-liver epigenetic patterns in the PDE groups, with correlation coefficients significantly exceeding those observed in the controls across developmental stages (*p* < 0.05, *p* < 0.01, Figure 6G–L). This cross-tissue coordination establishes PBMC H3K27ac as a potential biomarker for monitoring the hepatic cholesterol regulatory status.

#### 3.3.2. H3K27ac Level of LDLR in PBMC and Its Expression Negatively Related to the Blood Cholesterol Metabolic Phenotype in Male Newborns Received Antenatal Dexamethasone Treatment (ADT)

To investigate the predictive validity of the PBMC LDLR-H3K27ac epigenetic-transcriptional effect for fetal-originated hypercholesterolemia predisposition, we conducted validation using plasma samples from antenatal dexamethasone-exposed neonates and non-exposed controls. Standardized covariate analysis confirmed parity in maternal/neonatal baseline parameters (maternal age, gestational age, birth weight, and Apgar score) between cohorts (Table S1 in Supplemental Materials), establishing rigorous clinical comparability for subsequent biomarker evaluation.

**Figure 7:**
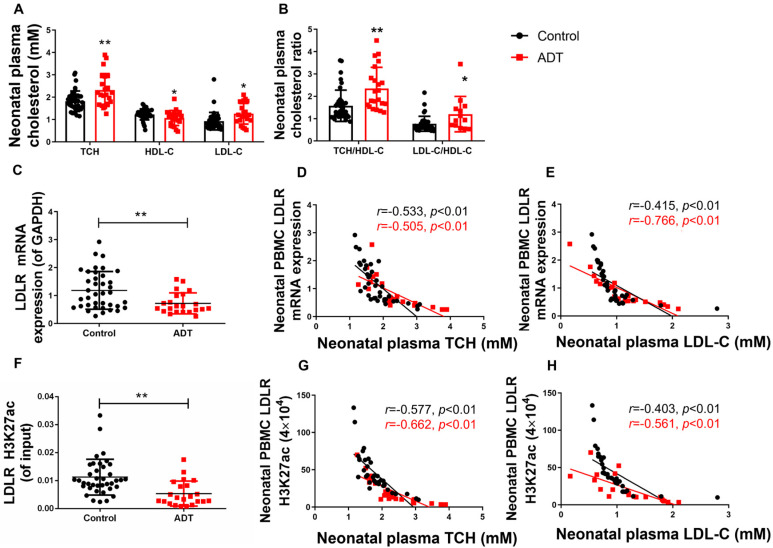
The low-density lipoprotein receptor (LDLR) histone 3 lysine 27 acetylation (H3K27ac) and mRNA expression levels in peripheral blood mononuclear cells (PBMC) and their correlation with the plasma total cholesterol/low-density lipoprotein-cholesterol (TCH/LDL-C) in male neonates who received antenatal dexamethasone treatment (ADT). (**A**) Plasma levels of TCH, high-density lipoprotein cholesterol (HDL-C), and LDL-C; (**B**) Plasma TCH/HDL-C and LDL-C/HDL-C concentration ratios; (**C**) PBMC LDLR mRNA expression (Black represents the Control group red represents the ADT group); (**D**) Correlation between PBMC LDLR expression and plasma TCH level; (**E**) Correlation between PBMC LDLR expression and plasma LDL-C level; (**F**) PBMC LDLR promoter H3K27ac level; (**G**) Correlation between PBMC LDLR promoter H3K27ac level and plasma TCH level; (**H**) Correlation between PBMC LDLR promoter H3K27ac level and plasma LDL-C level. *n* = 22 for the ADT group and *n* = 38 for the control group, mean ± SD. Intergroup comparisons were analyzed using Student’s *t*-tests, with 95% CIs for mean differences and effect sizes reported as Cohen’s d. * *p* < 0.05, ** *p* < 0.01 vs. control group.

A systematic evaluation of cholesterol profiles and PBMC biomarkers revealed significant dyslipidemia in ADT neonates. Compared with the controls, the ADT group exhibited elevated plasma TCH and LDL-C levels with reduced HDL-C levels (*p* < 0.05, *p* < 0.01, Figure 7A), resulting in significantly increased TCH/HDL-C and LDL-C/HDL-C ratios (*p* < 0.05, *p* < 0.01, Figure 7B). Concurrently, PBMC analysis revealed marked suppression of LDLR-H3K27ac levels (Cohen’s d = 0.79, 95%CI [0.15–0.78]) and mRNA expression (Cohen’s d = 1.02, 95%CI [0.003, 0.009]) (*p* < 0.01, Figure 7C,F). Correlation analysis demonstrated an inverse regulatory relationship between PBMC LDLR-H3K27ac/expression profiles and plasma TCH and LDL-C levels across both groups (*p* < 0.01, Figure 7D,E,G,H). These coordinated epigenetic-transcriptional alterations in circulating immune cells correlate with exacerbated cholesterol dyshomeostasis, establishing PBMC-based biomarkers as sensitive indicators of developmental metabolic reprogramming.

## 4. Discussion

### 4.1. PDE/ADT Can Induce Postnatal Hypercholesterolemia Susceptibility in Male Offspring

Current obstetrical guidelines mandate antenatal glucocorticoid receptor agonists (dexamethasone/betamethasone) as the standard intervention for preterm birth risk mitigation during gestational weeks 23–34, targeting fetal lung tissue maturation to prevent neonatal respiratory distress syndrome [15]. The National Institutes of Health (NIH) recommends intramuscular injection of dexamethasone at a dose of 6 mg every 12 h (cumulative 24 mg per course of treatment), equivalent to a 60 kg adult dose of 0.2 mg/kg [16,17]. The dose conversion between species was conducted through body surface area conversion (human:rat = 1:6.17), and 1.234 mg/kg was determined as the equivalent clinical dose for rats [18]. In conclusion, the 0.2 mg/kg dose used in the rodent model falls within the exposure range observed in human clinical applications. To simulate the human prenatal dexamethasone protocol, pregnant Wistar rats were subcutaneously injected with 0.2 mg/kg of dexamethasone on GD9-20 every day to establish a PDE rodent model. In the model we established, the dexamethasone concentration in fetal blood was quantitatively detected by HPLC to be 230 ± 61 nM [19]. Therefore, we set the concentration range for the in vitro experiment at 0–2500 nM, among which 500 nM was used as the concentration for the mechanism study of this experiment.

Hypercholesterolemia manifests as dysregulated lipid homeostasis, characterized by elevated TCH (TCH ≥ 5.20 mmol/L) or LDL-C (LDL-C ≥ 3.38 mmol/L) levels and/or reduced HDL-C (HDL-C ≤ 1.04 mmol/L) levels. Previous population surveys and animal experiments have revealed that prenatal metabolic insults predispose offspring to cholesterol dysregulation, which persists into adulthood [9,14,20,21,22]. Our PDE model demonstrated attenuated fetal plasma cholesterol fractions (TCH/HDL-C/LDL-C) in male offspring, consistent with prior findings [23] that dexamethasone exposure compromises placental sterol transporters, thus decreasing the amount of maternal-fetal cholesterol transfer. We further found that plasma TCH and LDL-C levels increased at PW8 (adolescence) and PW28 (adulthood) in male PDE offspring rats, leading to an increase in the ratios of serum TCH/HDL-C and LDL-C/HDL-C. Clinically, neonates with ADT demonstrate similar alterations in lipid profiles. In conclusion, PDE/ADT exposure predisposes male offspring to hypercholesterolemia and metabolic cardiovascular risks [14,24]. Notably, the more pronounced clinical neonatal phenotype compared to rodents may reflect human-specific postnatal compensatory mechanisms following intermittent antenatal drug administration, including enhanced cholesterol metabolic plasticity during developmental transitions.

### 4.2. The Suppressive Regulation of H3K27ac Level/Expression in the Hepatic LDLR Mediates Hypercholesterolemia in PDE Male Offspring

The liver regulates systemic cholesterol homeostasis through the dynamic regulation of sterol metabolic pathways. Transcriptional dysregulation of hepatic cholesterol-associated genes directly perturbs circulating lipid profiles, with LDLR pathogenic variants being mechanistically linked to atherosclerotic cardiovascular disease [25,26]. Emerging evidence positions H3K27ac, a histone modification marker associated with transcriptional activation, as a critical epigenetic mediator of in utero glucocorticoid-induced cholesterol dysregulation in offspring hepatocytes [22]. Building upon this molecular framework, we initially comprehensively screened hepatic cholesterol flux (biosynthesis, efflux, and reverse transport pathways) in male PDE offspring. The data revealed sustained suppression of hepatic LDLR expression across developmental stages (prenatal to postnatal), consistent with our previous mechanistic studies [8]. Histone acetylation site screening identified concomitant depletion of H3K27ac modifications at the LDLR promoter locus in male PDE offspring, with this epigenetic alteration exhibiting temporal synchrony with transcriptional downregulation throughout perinatal development. Our in vitro experiments confirmed a dose-dependent reduction in H3K27ac levels at the LDLR promoter in L02 cells by dexamethasone, accompanied by increased extracellular cholesterol levels. LDLR overexpression effectively reversed dexamethasone-induced abnormalities in cholesterol distribution. These results mechanistically link perinatal H3K27ac-LDLR dysregulation to the persistent hypercholesterolemia phenotype observed in male PDE offspring, suggesting the potential clinical utility of LDLR promoter H3K27ac as an epigenetic indicator of metabolic vulnerability.

Combined with previous studies, we analyzed and compared the sex differences in the mechanism of increased susceptibility to hypercholesterolemia in adult male and female offspring of PDE. The liver cholesterol reversal function (LDLR expression) of male PDE offspring continued to decrease, while the liver cholesterol synthesis function (HMGCR expression) of female offspring continued to increase, resulting in an increased susceptibility to hypercholesterolemia in adult offspring of different sexes. The sex difference in hypercholesterolemia between male and female adult offspring of PDE is related to the expression changes of liver sex hormone receptors [27].

### 4.3. Activated GR-Mediated the Suppressive Programming of Fetal Hepatocyte LDLR Expression Induced by Intrauterine Dexamethasone Exposure Through a Dual Pathway

Histone modification enzymes mediate histone modification of functional genes, which is a crucial mechanism underlying progeny programming changes in response to high intrauterine GC levels [28]. A systematic analysis of histone-modifying enzymes in PDE models revealed an exclusive elevation of hepatic HDAC2. Both in vivo and in vitro experiments demonstrated dexamethasone’s capacity to enhance HDAC2 expression and its promoter-specific association with the LDLR. Pharmacological HDAC2 inhibition using Santacruzamate A effectively reversed glucocorticoid-induced H3K27ac depletion, restored LDLR expression, and normalized cholesterol transport dynamics, establishing HDAC2-mediated epigenetic regulation as the principal mechanism underlying impaired reverse cholesterol transport.

**Figure 8:**
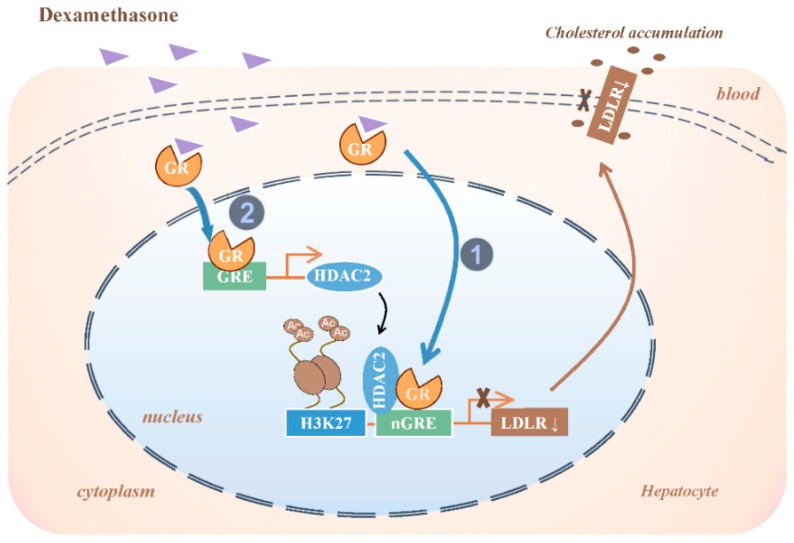
Molecular mechanism of decreased low-density lipoprotein receptor (LDLR) expression in fetal liver by prenatal dexamethasone exposure. GR, glucocorticoid receptor; GRE, glucocorticoid responsive element; nGRE, negative GC responsive elements; HDAC2, histone deacetylase 2; H3K27ac: histone 3 lysine 27 acetylation.

Dexamethasone mediates pharmacological effects through GR activation, which is a ligand-dependent nuclear receptor superfamily member. Activated GR regulates gene expression via two mechanisms: direct binding to glucocorticoid response elements (GRE/nGRE) in target promoters to modulate transcription [29], and combinatorial protein interactions with transcriptional co-regulators [30,31]. These synergistic actions coordinate glucocorticoid-driven cellular reprogramming, inducing persistent structural and functional adaptations [32]. Hepatic GR critically governs metabolic programming, with GR-mediated epigenetic dysregulation implicated in fetal-originated metabolic pathologies [9,33,34]. Our data reveal that dexamethasone-induced GR activation initiates two parallel pathways: direct binding to LDLR nGRE for transcriptional silencing and GR-dependent recruitment of HDAC2 to LDLR chromatin through both protein interaction and HDAC2 transcriptional induction. Pharmacological GR blockade (RU486) abolished these molecular events and restored physiological H3K27ac-LDLR regulation. This dual mechanism, which combines direct transcriptional repression and HDAC2-mediated epigenetic modulation, establishes a reinforcing circuit that exacerbates cholesterol transport impairment (Figure 8).

### 4.4. PBMC H3K27ac-LDLR Co-Downregulation Could Be the Predictive Epigenetic Signature for Developmental Hypercholesterolemia

Emerging evidence demonstrates that PBMC-derived epigenetic signatures and gene expression profiles reliably mirror tissue-specific molecular alterations, establishing their utility as non-invasive biomarkers [11,12,35,36]. Clinical observations have revealed synchronized circadian gene dysregulation in both PBMCs and ovarian granulosa cells in patients with PCOS and comorbid sleep disorders [35]. Mechanistically, PDE-induced H3K27ac depletion at SF1 promoter regions, observed concurrently in rat adrenal glands and neonatal PBMCs, underlies adrenal dysfunction in male offspring [12]. Prenatal bisphenol A exposure induces persistent epigenetic alterations, as evidenced by validated BDNF promoter hypermethylation in neonatal PBMC, which correlates with the development of autism spectrum disorder [11]. As circulating immune cells, the emerging role of PBMCs in metabolic regulation is underscored by their molecular concordance with hepatic lipid metabolism pathways, positioning them as viable surrogates for investigating cholesterol homeostasis [37,38]. Notably, the epigenetic nexus between PBMC histone acetylation dynamics and the developmental programming of cholesterol dysregulation remains unexplored in the current literature.

In this study, we discovered that adult male offspring of PDE rats were prone to hypercholesterolemia. Analysis of PBMC and liver tissues demonstrated consistent reductions in LDLR promoter H3K27ac levels and corresponding gene expression across developmental stages in the PDE groups, with a strong positive correlation between these parameters. Clinical validation using ADT-derived neonatal samples confirmed hypercholesterolemia at birth (characterized by elevated TCH and LDL-C levels) and the association between offspring plasma cholesterol profiles and PBMC epigenetic modifications. In neonates with PDE-associated hypercholesterolemia, PBMC LDLR promoter H3K27ac levels and expression were markedly reduced, showing negative correlations with circulating TCH and LDL-C concentrations. These findings suggest that diminished PBMC LDLR promoter H3K27ac levels and expression may serve as early biomarkers for hypercholesterolemia susceptibility in male PDE offspring, with disease conditions amplifying this association.

Early life is a critical period for organismal development, providing a valuable window for intervention in fetal-originated diseases due to its high developmental plasticity. Effectively preventing and treating fetal-origin metabolic diseases during early life and establishing early warning technologies are crucial, representing advanced concepts and hot topics in the current international field of fetal-origin disease research. Actively identifying biomarkers for the developmental toxicity of chemical exposure during pregnancy is vital for improving environmental health risk assessment systems. Animal studies have revealed that strategies regulating cholesterol-metabolizing enzymes through epigenetic mechanisms hold potential for prevention and treatment [39]. Recent studies have also suggested that exercise can reduce the risk of cardiovascular disease by improving cholesterol reverse transport function in rats (enhancing LDLR function) [40]. This indicates that the changes in LDLR epigenetic modifications in PBMCs proposed in our study can not only be used for disease risk monitoring in individuals at high risk for hypercholesterolemia, providing a new non-invasive monitoring tool for metabolic diseases [41], but may also become an effective environmental intervention target for potentially susceptible individuals. This new perspective opens up a new research direction for the early warning and intervention of health impacts by environmental pollutants, carrying significant scientific and public health implications.

## 5. Conclusions

In Summary, we demonstrated that PDE could induce hypercholesterolemia susceptibility in adult male offspring rats, and the intrauterine programming mechanism is related to the suppressed expression of fetal hepatocyte LDLR induced by dexamethasone activation of GR through a dual pathway (direct transcriptional inhibition and HDAC2/H3K27ac pathway). By combining animal and clinical models, we identified a strong concordance between PBMC LDLR promoter H3K27ac levels/expression patterns and the corresponding hepatic alterations. This study lays the groundwork for early warning technologies aimed at detecting cholesterol metabolism abnormalities during early life, facilitating the translation of fetal-originating disease research from basic studies to clinical applications. This study provides new theoretical and experimental evidence for environmental health risk assessment and personalized interventions.

## Data Availability

The data that support the findings of this study are available from the corresponding author upon reasonable request.

## Supplementary Materials

The following supporting information can be downloaded at: https://www.mdpi.com/article/10.3390/toxics13080651/s1, Figure S1: The schedule of animal treatment from gestational day (GD) 0 to postnatal week (PW) 28; Figure S2: Effects of prenatal dexamethasone exposure (PDE) on hepatic cholesterol metabolism in fetal and adult offspring rats. (A,D,G) mRNA expression of hepatic sterol regulatory element binding protein 2 (SREBP2), HMG CoA reductase (HMGCR), apolipoprotein B (ApoB), and scavenger receptor B1 (SR-B1) at gestational day (GD) 20, postnatal day (PW) 8, and PW28. (B,C,E,F,H,I) Protein expression of hepatic SREBP2, HMGCR, ApoB, and SR-B1 at GD20, PW8, and PW28. *n* = 12 for mRNA expression and chromatin immunoprecipitation (ChIP)-PCR; *n* = 3 for protein expression. Mean ± SD. Student’s *t*-test was used to compare the differences between the control and PDE groups. * *p* < 0.05, ** *p* < 0.01 vs. control group; Figure S3: Effects of prenatal dexamethasone exposure (PDE) on hepatic LDLR epigenetic modifications in fetal and adult offspring rats. (A–C) H3K9ac and H3K14ac levels in the LDLR promoter region at GD20, PW8, and PW28. *n* = 12 for mRNA expression and chromatin immunoprecipitation (ChIP)-PCR, *n* = 3 for protein expression. Mean ± SD. Student’s *t*-test was used to compare the control and PDE group differences. * *p* < 0.05, ** *p* < 0.01 vs control group; Table S1: Sources of chemicals and reagents; Table S2: Human and rat low-density lipoprotein receptor (LDLR) primers used for ChIP-PCR; Table S3: Characteristics of enrolled human subjects; Table S4: Human and rat primers used for real-time quantitative PCR.

## Abbreviations

PDE, dexamethasone during pregnancy; H3K27ac, histone 3 lysine 27 acetylation; LDLR, low-density lipoprotein receptor; GR, glucocorticoid receptor; HDAC2, histone deacetylase 2; PBMC, peripheral blood mononuclear cells; NCD, non-communicable diseases; GC, glucocorticoid; TCH, total cholesterol; LDL-C, low-density lipoprotein cholesterol; HDL-C, high-density lipoprotein cholesterol; MTS, 3-(4,5-dimethylthiazol-2-yl)-5-(3-carboxymethoxyphenyl)-2-(4-sulfophenyl)-2H-tetrazolium, inner salt; BCA, bicinchoninic acid; ChIP, Chromatin immunoprecipitation; ECL, Electrochemiluminescence; SR-B1, scavenger receptor class B type 1; HMGCR, HMG CoA reductase; SREBP2, sterol regulatory element binding protein 2; GAPDH, glyceraldehyde 3-phosphate dehydrogenase; H3K9ac, histone 3 lysine 9 acetylation; H3K14ac, histone 3 lysine 14 acetylation; IgG, immunoglobulin G; ApoB, apolipoprotein B; RU486, mifepristone; SPF, specific pathogen-free; GD, gestational day; PW, postnatal week; OD, optical density; GRE, Glucocorticoid responsive element; nGRE, negative glucocorticoid responsive element; ADT, antenatal dexamethasone treatment; NIH, National Institutes of Health; BDNF, brain-derived neurotrophic factor.
