# Supplementary material for: LDLR H3K27ac in PBMCs: An Early Warning Biomarker for Hypercholesterolemia Susceptibility in Male Newborns Treated with Prenatal Dexamethasone
Source: Toxics. 2025 Jul 31;13(8):651. doi: 10.3390/toxics13080651 (PMC12390111; doi:10.3390/toxics13080651)
Supplement: Supplementary file 1 [file toxics-13-00651-s001.zip › toxics-3727662-supplementary.pdf]

LDLR H3K27ac in PBMCs: An Early Warning Biomarker for Hypercholesterolemia Susceptibility in Male Newborns Treated with Prenatal Dexamethasone

Kexin Liu [1,2,†], Can Ai [1,†], Dan Xu [1,3], Wen Hu [2], Guanghui Chen [1], Jinzhi Zhang [1], Ning Zhang [1], Dongfang Wu [2,*] and Hui Wang [1,3,*]

[1] Department of Pharmacology, School of Basic Medical Science, Wuhan University, Wuhan 430071, China
[2] Department of Pharmacy, Zhongnan Hospital of Wuhan University, Wuhan 430072, China; huwen0113@163.com
[3] Hubei Provincial Key Laboratory of Developmentally Originated Disorder, Wuhan 430071, China

[*]Corresponding author:

Dongfang Wu

Department of Pharmacy, Zhongnan Hospital of Wuhan University, Wuhan 430072, China

E-mail: dfwu2010@whu.edu.cn

Hui Wang

Department of Pharmacology, School of Basic Medical Science, Wuhan University, Wuhan 430071, China

Mobile: +86-13627232557

E-mail: wanghui19@whu.edu.cn

Key words: dexamethasone; epigenetic inheritance; hypercholesterolemia; peripheral blood mononuclear cells; warning biomarker

Running title: LDLR H3K27ac: biomarker for hypercholesterolemia

SUPPLEMENTARY MATERIALS

1 Supplemental materials and methods

MTS assay

L02 cells were seeded at a density of 1 × 10^4 cells/well in 96-well plates. When the cells reached 80%–90% confluence, they were treated with dexamethasone (0, 100, 500, and 2500 nM) for 24 h. After having the medium of each well removed and washed twice with PBS, the cultures were incubated with 100 μL medium and 20 μL MTS at 37°C for another 2 h. The absorbance intensity was recorded at 490 nm using a microplate reader (Shimadzu, Kyoto, Japan), and the cell viability was normalized to a percentage relative to that of the untreated control cells.

TCH, HDL-C, and LDL-C concentration detection

The concentrations of TCH, HDL-C, and LDL-C in the plasma and cell samples were determined according to the manufacturer's protocols for the corresponding assay kits.

Total RNA extraction, reverse transcription, and RT-qPCR

Hepatic tissues or cultured cells were homogenized in TRIzol reagent. Total RNA was extracted according to the manufacturer's protocol. The concentration and purity of the isolated total RNA were determined using a spectrophotometer (NanoDrop 2000C, Thermo), and the RNA concentration was adjusted to 1 μg/μL. To convert total RNA into cDNA, single-strand cDNA was prepared from 1 μg of total RNA according to the protocol of the ExScript RT Reagent Kit. The primers used are listed in Table S1. All oligonucleotide primers for the experiments were custom-synthesized by Sangon Biotech Co. RT-qPCR was performed using an ABI Step One RT-PCR thermal cycler (ABI Stepone, NY, USA) in a 10 μL reaction mixture. To quantify the gene transcripts more precisely, the mRNA level of the housekeeping gene GAPDH was measured and used as a quantitative control. Each sample was normalized to GAPDH mRNA content. Relative amplicon expression was calculated using the $2^{-\Delta\Delta Ct}$ method.

Western blotting

Western blotting was performed according to the standard protocol. Rat liver tissues and L02 cells were lysed with RIPA lysis buffer. The protein concentrations of the samples were determined using a BCA protein assay kit. Nuclear protein fractions were obtained using the Nuclear-Cytosol-Mem Extraction kit. Rat liver tissues and cell lysates were mixed with 5 × loading buffer and heated for 10 min at 100°C. The proteins were separated by sodium dodecylsulfate polyacrylamide gel electrophoresis (SDS/PAGE) and then transferred onto immunoblot polyvinylidene difluoride membranes (Bio-Rad Lab., Hercules, CA, USA). After blocking, the proteins were incubated overnight at 4 °C with antibodies against LDLR (1:200 dilution), SREBP2 (1:200 dilution), HMGCR (1:1000 dilution), SR-B1 (1:2000 dilution), ApoB (1:1000 dilution), GR (1:1000 dilution), H3 (1:5000 dilution), and GAPDH (1:5000 dilution). After washing three times with Tris-buffered saline containing 1% Tween-20 (TBS-T), the membranes were incubated with a 1:5000 dilution of secondary antibodies for 1 h on an orbital shaker. After washing three times with TBS-T, the bands were visualized using an ECL chemiluminescent kit. A photo Documentation and Imaging System (BIO-ID VL, Conn, France) was used for densitometry and quantification of the protein bands.

Immunofluorescence analysis

Following the treatment, the cells were washed with ice-cold PBS three times, fixed in 4% formaldehyde, and blocked for 30 min with 3% BSA and 2% fetal bovine serum in 0.2% Triton X-100/PBS. The cells were then incubated overnight at 4°C with primary antibodies in blocking buffer, including mouse anti-HDAC2 (1:100 dilution) and rabbit anti-GR (1:200 dilution). The cells were washed with PBS and incubated with 1:200 diluted FITC-conjugated secondary antibody corresponding to anti-GR or anti-HDAC2 for 60 min at room temperature. Nuclei were stained with 4,6-diamidino-2-phenylindole (DAPI) at a 1:500 dilution for 5 min. The slides were washed twice with PBS. Negative controls obtained by omitting the primary antibodies showed

negligible background fluorescence. Fluorescence images of the transporters were captured using an immunofluorescence microscope (Nikon H550S, Tokyo, Japan). The quantitative fold change in transporter expression was analyzed using ImageJ version 1.44 (National Institutes of Health, Bethesda, USA).

Supplemental figure

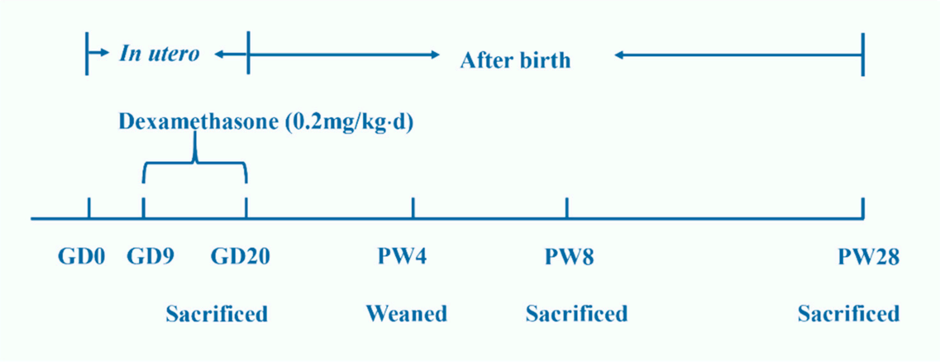

Figure S1. The schedule of animal treatment from gestational day (GD) 0 to postnatal week (PW) 28.

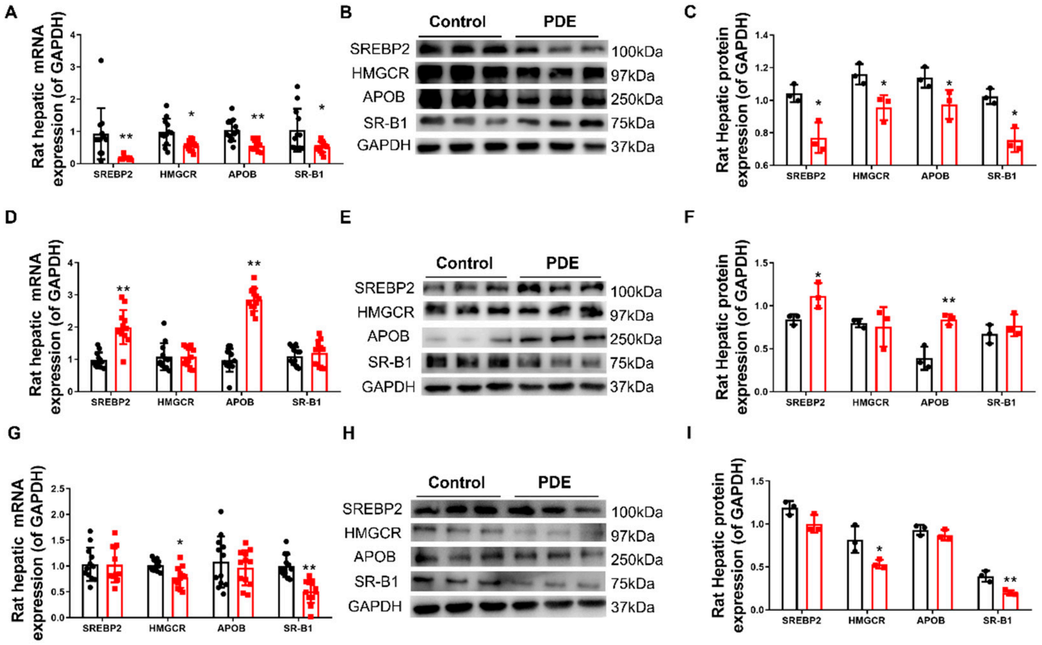

Figure S2. Effects of prenatal dexamethasone exposure (PDE) on hepatic cholesterol metabolism in fetal and adult offspring rats. (A, D, G) mRNA expression of hepatic sterol regulatory element binding protein 2 (SREBP2), HMG CoA reductase (HMGCR), apolipoprotein B (ApoB), and scavenger receptor B1 (SR-B1) at gestational day (GD)

20, postnatal day (PW) 8, and PW28. (B, C, E, F, H, I) Protein expression of hepatic SREBP2, HMGCR, ApoB, and SR-B1 at GD20, PW8, and PW28. n=12 for mRNA expression and chromatin immunoprecipitation (ChIP)-PCR, n=3 for protein expression. Mean ± SD. Student's t-test was used to compare the differences between the control and PDE groups. $^{*}P$<0.05, $^{**}P$<0.01 *vs* control group.

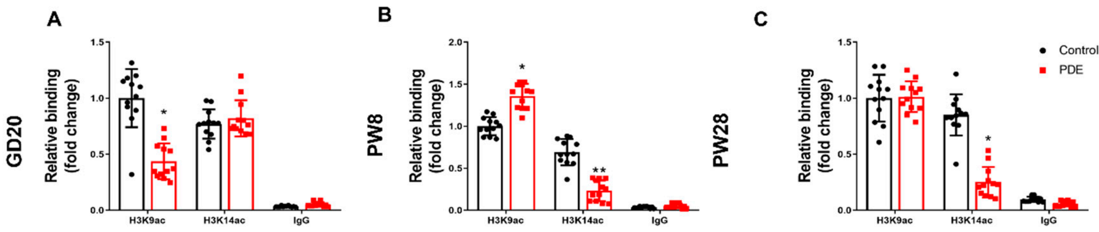

Figure S3. Effects of prenatal dexamethasone exposure (PDE) on hepatic LDLR epigenetic modifications in fetal and adult rat offspring. (A, B, C) H3K9ac and H3K14ac levels in the LDLR promoter region at GD20, PW8, and PW28. n=12 for mRNA expression and chromatin immunoprecipitation (ChIP)-PCR, n=3 for protein expression. Mean ± SD. Student's t-tests used to compare control and PDE group differences. $^{*}P$<0.05, $^{**}P$<0.01 *vs* control group.

Supplemental tables

Table S1. Sources of chemicals and reagents.

Reagents	Manufacturers
Magnoflorine	TargetMol (Boston, MA, USA)
Dexamethasone (No. 20140605)	Shuanghe Pharmaceutical Company (Wuhan, China)
Total cholesterol (TCH), low-density lipoprotein cholesterol (LDL-C), and high-density lipoprotein cholesterol (HDL-C) assay kits	Beijing Applygen Biotech Co., Ltd. (Beijing, China)
3-(4,5-Dimethylthiazol-2-yl)-5-(3-carboxymethoxyphenyl)-2-(4-sulfophenyl)-2H-tetrazolium, inner salt (MTS) assay kit	Cayman Chemical Co. (Ann Arbor, Michigan, USA)
oligonucleotide primers	Tianyihuiyuan Biotech Co., Ltd. (Guangzhou, China)
Reverse transcription and real-time quantitative polymerase chain reaction (RT-qPCR) kits	Takara Biotechnology Co., Ltd. (Dalian, China)
DNA purification kit	TIANGEN Biotechnology Co., Ltd (Beijing, China)
Chromatin immunoprecipitation (ChIP) assay kit	Millipore Co., Ltd. (Billerica, MA, USA)
Ficoll-Paque density gradient media	General Electric Company (Fairfield, Connecticut, USA)
Nuclear-Cytosol-Mem Extraction kit	Applygen (Beijing, China)

Bicinchoninic acid (BCA) protein assay kit	Beyotime (Shanghai, China)
Electrochemiluminescence (ECL) detection kit	Zhongshan Golden Bridge Biotechnology Co. Ltd. (Beijing, China)
Primary antibodies including glucocorticoid receptor (GR) (ab2768, ab183127), histone deacetylase 2 (HDAC2) (ab12169), LDLR (ab30532), scavenger receptor class B type 1 (SR-B1) (ab217318), HMG CoA reductase (HMGCR) (ab174830), sterol regulatory element binding protein 2 (SREBP2) (ab30682) and glyceraldehyde 3-phosphate dehydrogenase (GAPDH) (ab9485)	Abcam Co. (Cambridge, UK)
Antibodies of anti-histone 3 lysine 27 acetylation (H3K27ac) (A7253), anti-H3K14ac (A7254), anti-H3K9ac (A7255), goat anti-rabbit immunoglobulin G (IgG) (AC005), goat anti-mouse IgG (AC011), and apolipoprotein B (ApoB) (A4184)	ABclonal Technology Co., Ltd. (Wuhan, China)
H3 antibody (GB131012-1)	Biotechnology Inc. (Wuhan, China)
FITC-conjugated secondary antibody (GB22301)	Servicebio Inc. (Wuhan, China)
Mifepristone (RU486) (No. ODR4395) and proteinase K (No. ST533)	Kori Biotech Co., Ltd. (Wuhan, China)
Santacruzamate A (CAY10683) (1477949-42-C)	MedChemExpress, Ltd (Shanghai, China)
High-fat and high-fructose mouse chow	Xietong Pharmaceutical Bio-engineering Co., Ltd. (Jiangsu, China)
Dithiothreitol	Solabal Biotechnology Co. Ltd. (Beijing, China)
The plasmid LDLR PEX-4-no Neo vector (JP3949)	JTSBIO Co., Ltd (Wuhan, China)
GR overexpression plasmid and luciferase reporter plasmid	GenePharma Co., Ltd. (Suzhou, China)
Dual-Luciferase Reporter Assay System	Promega Co. (Madison, WI, USA)
Lipofectamine 3000	Invitrogen Co. (Carlsbad, CA, USA)

Table S2. Human and rat low-density lipoprotein receptor (LDLR) primers used for ChIP-PCR.

Genes	Forward primer (5’-3’)	Reverse primer (5’-3’)	Annealing
LDLR (human)	GTTCACGCCATTCTCCTGC	CACGAGGTCAGGAGATCAAGA	60°C, 30 s
LDLR (rat)	CGGTTTATGTGATGCTGGGG	AAGCCTGAGGACCTGAGTTC	60°C, 30 s

Table S3. Characteristics of the enrolled human participants.

**Indexes**	**Control (n=38)**	**ADT (n=22)**	**P value**
Maternal age (year)	31.74±0.86 (22-47)	32.55±0.10 (25-43)	N.S.
Gestational age (week)	34.40±0.27 (31-36.71)	34.03±0.34 (31.29-36.43)	N.S.
Birth weight (g)	2209±62 (1500-2910)	2120±111 (960-3250)	N.S.
Apgar scores (1 min)[a]	7.76±0.12 (6-9)	7.68±0.17 (6-9)	N.S.

Apgar scores (5 min)[a]	8.79±0.12 (7-10)	8.77±0.16 (7-10)	N.S.

Antenatal dexamethasone treatment (ADT). [a] Values are presented as median (interquartile range); N.Snot significant.

Table S4. Human and rat primers used for real-time quantitative PCR.

Genes	Forward primer (5'-3')	Reverse primer(5'-3')	Annealing
SR-B1 (human)	TTGATGCCCAAGGTGATG	CCTTATCCTTTGAGCCCTTT	60°C, 30 s
LDLR (human)	GAACCCATCAAAGAGTGCGG	CCACCCTCCAGGTTCACGCA	60°C, 30 s
ApoB (human)	AGGCATCTCCACCTCAGCAG	GGAGAGGACTGAGGGCTGTAGT	60°C, 30 s
HMGCR (human)	GTAGAGGAAGAGACAGGGATAA	CGAGTAAGGAGGAGTTACCA	63°C, 30 s
GAPDH (human)	CAAGAGCACAAGAGGAAGAG	GTTGAGCACAGGGTACTTTA	63°C, 30 s
GR (human)	TTCTGACTGGGGCCAATGAA	AATGATGGTGGCCTCGAGA	60°C, 30 s
HDAC2 (human)	ATGGCGTACAGTCAAGGAGG	TGCGGATTCTATGAGGCTTCA	63°C, 30 s
SREBP2 (human)	GCAGTCTGGTGGACAATGAGG	TCATCCAATAGAGGGCTTCC	60°C, 30 s
HMGCR (rat)	CTGGTGAGTTGTCCTTGATG	CCGTGTTTCAGTCCAGTATG	60°C, 30 s
SR-B1 (rat)	CTTCTGGTGCCCATCATTTA	CCTACAGCTTGGCTTCTTG	63°C, 30 s
LDLR (rat)	GGATCCATGGCAACATCTAC	ACCCTTTCTCTCGGAACA	63°C, 30 s
ApoB (rat)	ATGGTGCATGAGTATGCCAA	ATCCCACTTTTGGGAGGTTC	60°C, 30 s
GR (rat)	CTGTGTTCACTTTCTGAAGCCAT	CCCAGGCATTGCTCTTTGAT	60°C, 30 s
GAPDH (rat)	GCAAGTTCAACG GCACAG	GCCAGTAGACTCCACGACA	60°C, 30 s
HDAC2 (rat)	GGACAAGAGGACAGATGTTAAGG	GGGTTGTTGAGTTGTTCTGATTT	60°C, 30 s
SREBP2 (rat)	AGGTCTAGGGATGGGTGAAA	GTGGGAAGGAACAGGACAATTA	63°C, 30 s

SR-B1, scavenger receptorB1; LDLR, low-density lipoprotein receptor; ApoB, apolipoprotein B; HMGCR, HMG CoA reductase; GAPDH, glyceraldehyde 3-phosphate dehydrogenase; GR, glucocorticoid receptor; HDAC2, histone deacetylase 2; SREBP2, sterol regulatory element binding protein 2.
